# An Investigation on the Effects of Ship Sourced Emissions in Izmir Port, Turkey

**DOI:** 10.1155/2013/218324

**Published:** 2013-10-01

**Authors:** Halil Saraçoğlu, Cengiz Deniz, Alper Kılıç

**Affiliations:** ^1^Vacational High School, Istanbul Technical University, Maslak, 34469 Istanbul, Turkey; ^2^Department of Marine Engineering, Istanbul Technical University, Tuzla, 34940 Istanbul, Turkey

## Abstract

Maritime transportation is a major source of climate change and air pollution. Shipping emissions cause severe impacts on health and environment. These effects of emissions are emerged especially in territorial waters, inland seas, canals, straits, bays, and port regions. In this paper, exhaust gas emissions from ships in Izmir Port, which is one of the main ports in Turkey, are calculated by the ship activity-based methodology. Total emissions from ships in the port is estimated as 1923 ton y^−1^ for NO_*x*_, 1405 ton y^−1^ for SO_2_, 82753 ton y^−1^ for CO_2_, ton y^−1^ for HC, and 165 ton y^−1^ for PM in the year 2007. These emissions are classified regarding operation modes and types of ships. The results are compared with the other studies including amounts of exhaust pollutants generated by ships. According to the findings, it is clear that the ships calling the Izmir Port are important air polluting causes of the Izmir city and its surroundings.

## 1. Introduction

The most important impacts of air pollution are climate change, reduction of ozone layer thickness, acid rains, and the corruption of air quality. One of the most significant air pollution sources are ship-generated emissions. Maritime transportation is the major transportation mode as in that the international marine transport of goods is responsible for roughly 90% of world trade by volume [1]. Similarly, more than 80% of world trade is carried by sea in terms of weight [2]. The world maritime fleet has grown in parallel with the seaborne trade registered under the flags of over 150 nations [3]. 

Over the past decades, growing international trade resulted in a corresponding growth in the tonnage of merchandise carried by ships [4]. The merchant shipping industry and the development of the world economy are closely related [5]. Maritime transportation is considered to be the most energy efficient cargo transportation mode, which has the potential to make a significant contribution to the efficiency of the transport system. 

The growing number of shipping movements and the related release of air pollutants have drawn attention onto this emission source. Shipping activities are one of major air pollution sources as the ships that have high powered main engines often use heavy fuels. More than 95% of the world's shipping fleet is powered by diesel engines [6].

Since the shipping emissions have not been controlled tightly, there some difficulties to achieve progress in improving environmental performance. Because their air pollutant emissions remain comparatively unregulated, ships are now among the world's most polluting combustion sources per ton of fuel consumed [7]. The bunker oil used in ocean going ships has been estimated to produce over 100 times compared to on-road diesel per unit volume [8]. Ship emissions have remarkable global, regional, and local adverse impacts on the air quality on sea and land. The most important pollutants emitted from ships are nitrogen oxide (NO_*x*_), sulfur dioxide (SO_2_), carbon dioxide (CO_2_), hydrocarbons (HC), and particulate matter (PM). Shipping emissions are easily transferred long distances in the atmosphere from the sea the land and between the continents [9]. Also, the effects of shipping emissions can increase in the domestic seas, narrow channels, straits, gulfs, and port areas specially including dense maritime traffic, sensitive ecosystems and the presence of populations. The health effects of air pollution at ports may include asthma, other respiratory diseases, cardiovascular disease, lung cancer, and premature death [10].

Significant progress in estimating international ship emissions has been made in the past decade. Furthermore several global, regional, and local inventory studies have been performed. The emissions of NO_*x*_, SO_2_, PM, and GHG's (Green House Gases) from global shipping are increased from 585 to 1096 million tons between 1990–2007 [11]. The CO_2_ emissions from international shipping are estimated at 943.5 million tons for the year 2007 [12]. According to a report by TRT (2007), CO_2_ emissions from global shipping are about 1 billion tons for the year 2006 [13]. International shipping is responsible for 3% of global CO_2_ emissions (11). Based on the fuel consumption, the annual CO_2_, NO_*x*_ and SO_*x*_ emissions from ship corresponds to about 2%, 11%, and 4% of the global anthropogenic emissions, respectively [14]. 

The port areas are the most recognizable receptors of pollutants emitted from ships. The emissions from ships may threaten the air quality while berthing or maneuvering and in coastal communities while transiting along the coast. Approximately 80% of the world fleet are either harbored (55% of the time) or near a coast (25% of the time) [1]. This means that ships spend about 20% of the time far from land [7].

There are many local studies about estimating the shipping emissions in gulfs and port regions in the literature. It was estimated that the shipping emissions were approximately 1.725 Mt NO_*x*_, 1.246 Mt SO_2_, 0.147 Mt CO, and 0.035 Mt HC in the Mediterranean Sea and the Black Sea regions based on ship movements [15]. The International Institute for Applied Systems Analysis (IIASA) estimated that the shipping emissions of CO_2_, NO_*x*_, SO_2_, and HC were 77.140 Mt, 1.818 Mt, 1.278, and 0.062 Mt, respectively, in the Mediterranean Sea [16]. The shipping emissions in the Black Sea were estimated at 3.85 Mt of CO_2_, 0.089 Mt for NO_*x*_, 0.065 for SO_2_ [16]. Deniz and Durmuşoğlu carried out to define as 0.11 Mt for NO_*x*_, 0.087 Mt of SO_2_ in the Sea of Marmara [17]. Minjiang et al. carried out to characterize the air pollutants in Shanghai Port and identify the contribution from ship traffic emission [18]. Tzannatos, estimated the shipping emissions and externalities for Port of Piraeus [19]. The shipping emissions were estimated by Saxe and Larsen (2004) for three Danish ports, Kılıç and Deniz (2010) for Izmit Gulf-Turkey, Deniz and Kilic (2010) for Ambarli Port, Deniz and Kilic (2010) for Candarli Gulf [20–22].

In this study, the shipping emissions are calculated based on the real shipping activities and engine power information for Izmir Port-Turkey as a major export port region of the country. The annual emissions from ships are calculated as 1923 t y^−1^ for NO_*x*_, 1405 t y^−1^ for SO_2,_ 82753 t y^−1^ for CO_2_, 74 t y^−1^ for HC, and 165 t y^−1^ for PM.

## 2. Location and Time of Study

**Figure 1:**
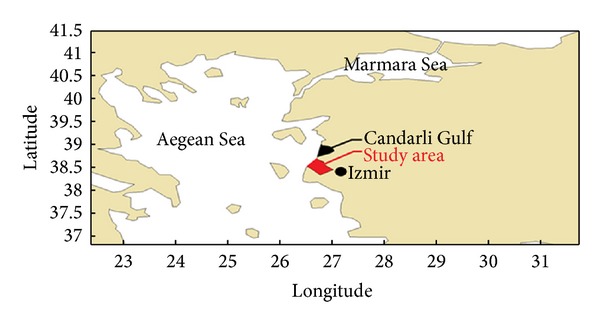
Study Region-Izmir Gulf.

The Izmir Port, one of the important export ports in Turkey, plays a vital function for the Aegean Region's industrial and agricultural experts. Izmir port is the biggest container terminal and has a great logistic importance for the Turkish economy. Also, it is a trading center because of an increment on the port capacity in the years. The study region is illustrated in Figure 1.

It is the only container handling terminal in this region and has 559.661 TEU and 9.652.714 ton cargo handling capacity per year. In addition, the port has the capacity to accommodate 3.640 ships per year. The port is also one of the largest passenger port in Turkey because Izmir is a tourism center and because of the surrounding historical places to visit.

In 2007, 2803 vessel arrivals, 12 million tons of cargo being handled, and 300.000 passengers pass through the port. The port is also connected with state railway and highway network. In 2008, 11 million tons cargo was handled at Izmir Port; therefore, this amount corresponded to %37 of all cargos handled at other Turkish ports.

Ship fleet information acquired from unique ship records is indicated in Table 1. The number of General Cargo ships consists of 60% of all vessels which followed by Container ships with 30%. Since some vessels call at port more than once and berthing time characteristics of the port depend on port productivity of each ship call, berthing time statistics were calculated based on each ship calls where the other particulars reflects the unique ship characteristics. As a result, the significant number of container ships call in Izmir port constitutes 56% of all ships, while general cargo ships make up 35% of all calls. Statistics based on ships calling into Izmir port were evaluated in the year 2007.

## 3. Methodology 

Ship emissions were calculated by the ship activity-based method which involves the application of emission factors for each ship-activity (cruising, maneuvering, and hotelling). The emission factors are critically important to determine representative values of ship emissions for the ship's engines during that activity. Furthermore, emission factors depend on speed of the ship and the fuel type. 

**Figure 2:**
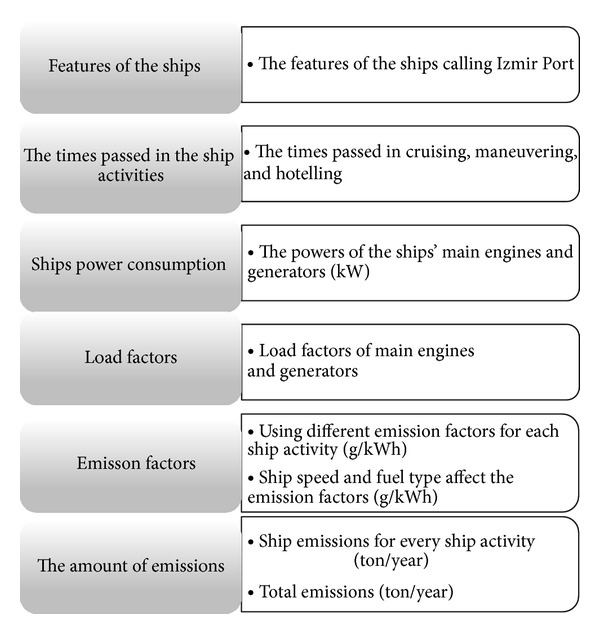
The flow chart for the used ship activity-based method.

Ship activity-based method was used to estimate the ship emissions in Izmir port. This method is clarified by flow charts and illustrated in Figure 2. The ship activity-based methodology was applied to the ships calling the Izmir Port to estimating the amounts of the main ship exhaust pollutants (NO_*x*_, SO_2_, CO_2_, HC, and PM) while cruising, maneuvering and hotelling. Ship emissions depend on the time passed in the ship activities, ship power consumption, emission factors, load factors of main engines, and generators. 

The exhaust gas emissions were calculated for 2803 ships called Izmir Port in 2007. The emissions produced during the ship's cruising, maneuvering, and hotelling were estimated through the application of the following expressions [23]:

(1) ECruising(g)  =DV[ME·LFME·EF1+AE·LFAE·EF1],EManeuvering(g)=TManeuvering(ME·LFME·EF2       +  AE·LFAE·EF2),EHotelling(g)=THotelling(AE·  LFAE·  EF3),

where *ME* is a main engine power (kW), *AE* is a generator power (kW), *V* is a ship average speed between cruising and maneuvering (km/h), *D* is a distance between cruising and maneuvering (km), *LF*_*ME*_ is a load factor of main engine at cruising, maneuvering and hotelling (%), *LF*_*AE*_ is a load factor of generator at cruising, maneuvering and hotelling (%), *EF*_1_ is an emission factors for cruising mode (g/kWh), *T*_Man_ is an average time spent during maneuvering (h), *EF*_2_ is emission factors for maneuvering mode (g/kWh), *T*_Hotelling_ is an average time spent at berth (h), and *EF*_3_ is an emission factors for hotelling (g/kWh).

The load factors of the main engine and auxiliary engines for cruising, maneuvering and hotelling modes are illustrated in Table 2. 

Total cruising distance in the gulf is 128.8 km. The cruising times of ships were determined based on the ship's default service speed at 80% MCR. Since the main engine load is assumed as %40, the half of the service speed of the vessels is used. Ships default service speeds are shown in Table 3 [24]. The cruising ship emissions were calculated for each ship's one main engine and two numbers of generators. At cruising mode, main engine loads were assumed as 40% instead of 80% because of the structure of the gulf. Also, for the ship's safety, at cruising mode, it is estimated that the ships operate two generators synchronized. 

Maneuvering emissions are calculated for each ship's one main engine and two parallel generators. During maneuvering, main engine load decreases so load factor in this mode declines to 40% [23]. The average time for maneuvering is a total 2 hours including arrival and departure, obtained by Under Secretariat for Maritime Affairs [25]. 

It is assumed that the main engine is stopped and one generator is running while loading and unloading the cargo at berthing. Main Engine (ME) load is assumed as 20% and percentage of main engine operation time is assumed as 5%. There is one generator running which load factor is 40% at hotelling phase. The emission factors are shown in Table 4 [23, 24]. The berthing time for each ship calls were obtained from Under secretariat for Maritime Affairs [25].

The data used to estimate ship exhaust emissions as main engine powers, generator powers and ships duration time in the berth, are the actual values for the ships calling the Izmir Port. Since the engine power, engine load, and engine running hours are the key factors to estimate the emissions, using the exact values of these data gives more accurate results. 

**Figure 3:**
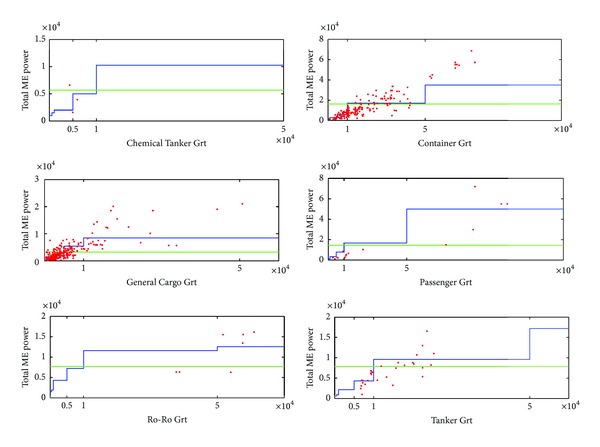
Comparison of ME Powers with Default.

The significant data of main engine and generator powers of the ships called Izmir Port are explored at Lloyds Register ship data bank [24]. ME powers of ships are compared to the default values of literature which are classified by ship type and ships gross tonnage (Figure 3) [26]. It is obvious that, linear function could be more appropriate instead of stair function especially above and higher than 50 thousand gross tonnages of container ships and 10 thousand gross tonnages of general cargo ships.

## 4. Results and Discussion

In this study, the exhaust emissions are calculated with the activity-based emission model for the Izmir Port, which is the most important container port in Turkey. It is determined that ships calling into Izmir Port are a major source of air pollutants in the city of Izmir. Also, it is stated that ship emissions may lead to critical effects upon human health because Izmir port is within the city of Izmir, which has the third highest population of Turkey. 

**Figure 4:**
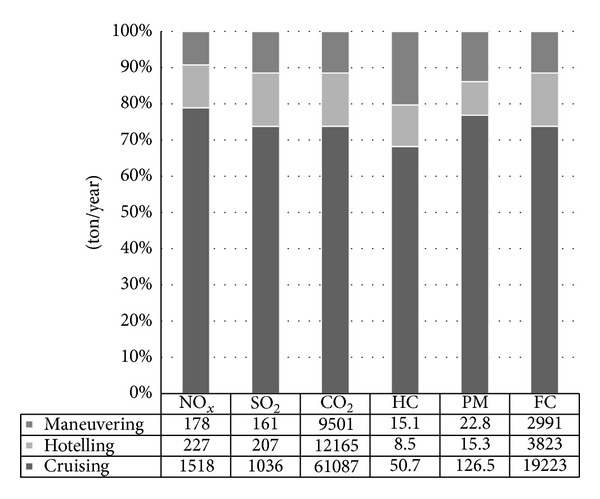
Total exhaust emissions during ship operational modes.

As seen from Figure 4, the amounts of emissions during ship operations were 1923 t y^−1^ for NO_*x*_, 1405 t y^−1^ for SO_2_, 82753 t y^−1^ for CO_2_, 74 t y^−1^ for HC, and 165 t y^−1^ for PM. Approximately 26000 tons of fuel were consumed in the gulf by the ships. The emissions during cruising mode were higher than maneuvering and hotelling emissions due to longer distances, also the main engine and one generator were operated at the maximum load. Ship emissions released during hotelling, maneuvering, and cruising modes are illustrated in Figure 4. The exhaust gas pollutants generated from ships during cruising were 66.8% of the total amounts in operational modes. Moreover, while maneuvering emissions were 18.1% and during hotelling 15.1% of all amounts.

**Figure 5:**
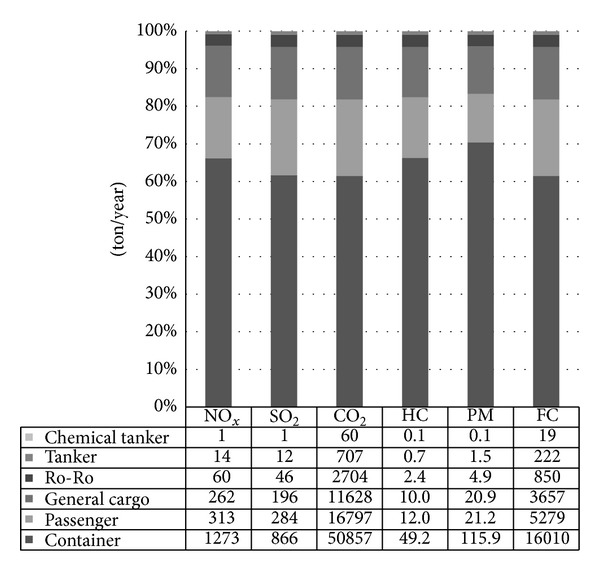
Total exhausts emissions according to ship types.

Also exhaust gas emissions according to ship types are specified in Figure 5. The highest levels of exhaust gas emissions were generated from container ships. General cargo and cruise ships also emit large amounts of exhaust gas as seen in the dataset. 

The percentage of NO_*x*_ emissions is shown in Table 5. Container ships constitute 66% of all NO_*x*_ emissions at all operating modes and 74% of all NO_*x*_ emissions generated by ME by ships at cruising modes. Each cell contains two percentage ratios; the first one indicates the emission amount ratio of ship type whilst and the second shows the engine and operating mode ratio of a certain ship type. The multiplication of these values of each cell gives the overall ratio of specified engines at operation modes of a given ship type. For instance, at hotelling mode auxiliary generators of general cargo ships generates 5.76% (0.32 × 0.18) of all NO_*x*_ emissions.

Within the city of Izmir, the air pollutant-emitting sources may be divided into land- and ship-based sources. Land-based sources for an air pollutant is domestic heating, traffic, and industry for Izmir city. Land-based emissions are compared to annual shipping emissions in Izmir Port in the Table 6. 

The land-based sources of air pollutants within Izmir city was found as 23,173 t of NO_*x*_, 13,094 t of SO_2_ and 16,451 t of PM [27]. 

The shipping emissions in Izmir Port are compared with other specific ports in in Table 7. SO_2_ emissions from ships calling at Izmir Port have the most amounts because of the higher content of sulfur in marine fuels.

The NO_*x*_ and SO_2_ emissions from ships in Izmir port are more than those of other ports except Oakland Port. Furthermore, ship emissions are compared between Izmir Port and other Turkish Ports in the Table 8. The amount of exhaust gas emissions from ships calling into Izmir Port is the second highest amount except ships calling into Izmit Gulf. 

## 5. Conclusion

Ship emissions are a significant source of air pollution in cities and have a direct effect on the human population. In this study, the estimation of exhaust gas emissions (NO_*x*_, SO_2_, CO_2_, HC, and PM) from ships in Izmir Port is calculated on the shipping activity based bottom up approach for the first time. The annual emission rates are calculated as 1923 ton y^−1^ for NO_*x*_, 1405 ton y^−1^ for SO_2_, 82753 ton y^−1^ for CO_2_, ton y^−1^ for HC, and 165 ton y^−1^ for PM.

The emissions generated from ships calling into Izmir port might have critical health effects on people living close to Izmir which has the third highest population of Turkey. Some precautions can take to decrease the ship emissions in the port. Most of the emissions are released during cruising and hotelling of ships. The cold ironing method could be used for electrical energy demands of the ships to cut off hotelling emissions. All emissions near the port should be monitored regularly.

This paper presents the first ship emission inventory to estimate the ship emissions for Izmir port. Consequently, the ships calling the Izmir Port are important air polluting sources of the Izmir city and its surroundings. The result will help next studies to compare and observe the ship emission inventories for Izmir port. As a conclusion, collected data and results can be used in estimating ship exhaust emissions studies for Izmir.

## Figures and Tables

**Table 1:** Ship Particulars at Izmir Port for the year 2007.

	Number of ships		Max	Min	Average	Median	Std Dev.
Chemical	3	GRT	5998	4358	5115	4989	827
		ME kW	6564	1560	4008	3900	2504
		ME rpm	580	210	444	542	204
		DG kW	330	300	310	300	17
	4	Berth Time	91	30	65	69	25

Container	260	GRT	75590	959	19055	14821	14601
		ME kW	68470	550	13592	10130	11960
		ME rpm	960	65	254	127	217
		DG kW	1000	100	458	440	161
	1567	Berth Time	120	1	21	19	11

General cargo	502	GRT	50681	393	4262	2531	5406
		ME kW	21000	170	2650	1609	3036
		ME rpm	1200	79	538	500	258
		DG kW	1000	50	272	245	137
	976	Berth Time	376	3	39	28	37

Passenger	19	GRT	114147	2889	52014	22080	46874
		ME kW	72000	1200	25517	10294	26239
		ME rpm	750	78	450	450	209
		DG kW	800	200	531	525	220
	141	Berth Time	61	4	10	8	8

Ro-Ro	16	GRT	60942	37710	47168	51714	8751
		ME kW	60942	37710	47168	51714	8751
		ME rpm	113	100	111	112	3
		DG kW	1180	310	674	500	249
	81	Berth Time	36	3	13	13	6

Tanker	30	GRT	25487	6650	13955	11450	6474
		ME kW	16550	1030	6727	6480	3260
		ME rpm	950	102	235	140	213
		DG kW	750	200	406	400	149
	34	Berth Time	113	19	43	41	21

All Ships	830	GRT	114147	393	11169	4968	15712
		ME kW	72000	170	6911	3150	9962
		ME rpm	1200	65	427	450	279
		DG kW	1180	50	349	330	181
	2803	Berth Time	376	1	27	20	25

**Table 2:** Load factors of main engine and generators according to operational modes.

Operational mode	Main engine load	Generator load
Cruising	%40	%30
Maneuvering	%40	%50
Hotelling	%20	%40

**Table 3:** Average ship speed of the ships called Izmir Port.

Ship type	Ship speed (km/h)
Chemical tanker	27.78
Container	37.04
General cargo	25.93
Passenger	37.04
RO-RO	33.34
Tanker	25.93

**Table 4:** Emission factors used in the calculation (g/kWh).

Ship types	NO_*x*_			SO_2_			CO_2_			HC			PM			SFC		
	Cru	Hotel	Man	Cru	Hotel	Man	Cru	Hotel	Man	Cru	Hotel	Man	Cru	Hotel	Man	Cru	Hotel	Man
Chemical T.	16.3	13.3	13.3	11.0	12.2	12.2	650	716	715	0.55	1.00	1.04	1.34	1.50	1.60	204	225	225
Container	17.3	13.5	13.8	10.8	12.3	12.0	635	720	705	0.57	0.50	1.19	1.56	0.90	1.73	200	226	222
Gen. Cargo	16.2	13.4	13.2	10.9	12.2	12.1	649	721	715	0.54	0.50	1.03	1.28	0.90	1.59	204	227	225
Passenger	13.2	13.2	11.8	11.8	12.3	12.6	697	725	747	0.46	0.50	0.97	0.81	0.90	1.71	219	228	235
Ro-Ro	15.3	13.3	12.8	11.1	12.3	12.2	655	722	719	0.52	0.50	1.06	1.17	0.90	1.68	206	227	226
Tanker	14.8	12.5	12.5	11.7	12.6	12.7	690	743	745	0.50	1.10	1.10	1.43	1.70	1.82	217	234	235

**Table 5:** NO_*x*_ percentage according to ship type and operation mode.

Ships	Percentage of NO_*x*_						Total
	Cruising		Maneuvering		Hotelling		
	ME	AE	ME	AE	ME	AE	
Chemical tanker	0-72	0-9	0-7	0-3	0-2	0-7	0-100
Container	67-75	59-4	72-8	64-2	69-4	58-7	66-100
General cargo	11-60	29-10	10-6	23-3	15-5	32-18	14-100
Passenger	19-85	5-1	14-6	6-1	14-4	7-3	16-100
Ro-Ro	3-74	5-7	3-9	5-3	2-2	2-5	3-100
Tanker	1-67	1-9	1-8	1-2	1-4	1-10	1-100
All ships	100-74	100-4	100-8	100-2	100-4	100-8	100-100

**Table 6:** Land-based emissions in (t y^−1^).

Air pollutant sources	NO_*x*_	SO_*x*_	PM
Domestic heating	1.124	5.693	11.159
Traffic	19.418	1.862	1.351
Industry	2.631	5.539	3.941
Shipping	1.923	1.405	165

**Table 7:** Comparison of shipping emissions on the different ports (t y^−1^).

Port	Ships call	NO_*x*_	SO_2_	HC	PM	Source
Aberdeen	—	376	52	—	14	[28]
Copenhagen	—	743	162	—	13	[20]
Oakland	1.916	2.484	1.413	—	219.5	[29]
JN-New Bombay	2.900	397	56	—	221	[30]
Port Arthur	—	1716	833	—	133	[31]
Izmir	2.806	1.923	1.405	74	165	In this study

**Table 8:** Shipping emissions at Turkish ports (t y^−1^).

Turkish ports	Ships call	NO_*x*_	SO_2_	CO_2_	PM	Source
Izmit Gulf	11.645	5.356	4.305	254.261	232	[21]
Ambarlı Port	5.432	845	242	78.590	36	[22]
Çandarlı Gulf	7.520	632	574	33.848	32	[6]
Izmir Port	2.806	1.923	1.405	82.753	165	This study
